# Association of Prenatal Alcohol Exposure and Offspring Depression: A Negative Control Analysis of Maternal and Partner Consumption

**DOI:** 10.1111/acer.14324

**Published:** 2020-04-21

**Authors:** Kayleigh E. Easey, Nicholas J. Timpson, Marcus R. Munafò

**Affiliations:** ^1^ UK Centre for Tobacco and Alcohol Studies School of Psychological Science University of Bristol Bristol UK; ^2^ MRC Integrative Epidemiology Unit University of Bristol Bristol UK; ^3^ Bristol Medical School University of Bristol Bristol UK

**Keywords:** Alcohol, Pregnancy, Depression, Avon Longitudinal Study of Parents and Children

## Abstract

**Background:**

Previous research has suggested that intrauterine alcohol exposure is associated with a variety of adverse outcomes in offspring. However, few studies have investigated its association with offspring internalizing disorders in late adolescence.

**Methods:**

Using data from the Avon Longitudinal Study of Parents and Children (ALSPAC), we investigated the associations of maternal drinking in pregnancy with offspring depression at age 18 and 24 (*n* = 13,480). We also examined partner drinking as a negative control for intrauterine exposure for comparison.

**Results:**

Offspring of mothers that consumed any alcohol at 18 weeks gestation were at increased risk of having a diagnosis of depression (fully adjusted model: OR 1.17, 95% CI 1.02 to 1.34), but there was no clear evidence of association between partners’ alcohol consumption at 18 weeks gestation during pregnancy and increased risk of offspring depression (fully adjusted model: OR 0.87, 95% CI 0.74 to 1.01). Postestimation tests found a positive difference between the association of maternal and partner alcohol use on offspring depression, showing a stronger association for maternal compared with partner alcohol use (OR 1.41, CI 1.07 to 1.84).

**Conclusions:**

Maternal drinking in pregnancy was associated with increased risk of offspring depression at age 18. Residual confounding may explain this association, but the negative control comparison of paternal drinking provides some evidence that it may be causal, and this warrants further investigation.

Alcohol consumption during pregnancy is common, with 40 to 80% of expectant mothers in Australia, New Zealand, and the UK (O'Keeffe et al., 2015), reporting consuming alcohol when pregnant. This high percentage of women reporting alcohol use may be in part due to previous guidelines, which suggested that low levels of consumption are safe for the developing fetus. Until recently in the UK, for example, guidelines advised pregnant women to abstain from alcohol in the first 3 months of pregnancy; however, these guidelines also stated that there is no evidence that a low level of alcohol use of 1 to 2 units (2 units being a 175‐ml glass of wine), no more than once or twice a week, is linked to harm in the unborn child (Nice, 2008). Guidelines for alcohol use during pregnancy have only recently been updated to advise that women should abstain from alcohol consumption during their entire pregnancy (Department of Health, 2016). This change is due in part to growing evidence that maternal alcohol consumption in pregnancy is associated with several negative health outcomes in offspring.

It is well established that heavy alcohol use in pregnancy can cause fetal alcohol syndrome (Mukherjee, Hollins and Turk, 2006), resulting in physical and cognitive impairments (Coles et al., 2002; Gibbard, Wass and Clarke, 2003; Guerri, Bazinet and Riley, 2009). However, even at levels of alcohol consumption below that required for fetal alcohol syndrome, exposure to alcohol during gestation has been shown to be associated with detrimental outcomes in the offspring, such as being small for gestational age (Mamluk et al., 2017) and birth complications such as preeclampsia and placental abruption (Salihu et al., 2011), as well as behavioral outcomes such as increased risk of externalizing disorders (Sayal et al., 2014) and internalizing disorders (Easey et al., 2019; Sood et al., 2001; Walthall, O'Connor and Paley, 2008). However, much research in this area has been conducted on offspring at an early age, with less research conducted on older age‐groups to establish whether these associations persist into adulthood. One of the few studies to have used an older offspring age‐group suggested that the detrimental outcomes shown for gestational exposure to alcohol are likely to be permanent as they were still evident at age 22 (Day et al., 2013), although replication of this finding is required. Low levels of intrauterine alcohol exposure have also been shown to be protective against offspring internalizing and externalizing problems in some studies, suggesting that residual confounding may influence observed associations (Kelly et al., 2009; Robinson et al., 2010b).

Frequency, pattern, and timing have also been shown to be important when investigating maternal alcohol use in pregnancy, as opposed to just the presence or absence of consuming alcohol (O'Leary et al., 2010). Day and colleagues (2013) reported a dose–response association for alcohol use during pregnancy across all 3 trimesters with increased offspring mental health problems. However, the evidence is mixed for specific associations during different trimesters. Niclasen and colleagues (2014a) reported evidence that binge drinking at both 16 and 30 weeks gestation was associated with conduct disorder. On the other hand, O’Leary did not find evidence of an association with internalizing disorders when their analyses were restricted to the third trimester (O'Leary et al., 2009).

Observational studies such as these can identify associations. However, the well‐described problems of bias, reverse causation and confounding, are likely responsible for conflicting evidence on the effects of intrauterine alcohol exposure, and causal inference is difficult. Mendelian randomization (MR) is a method used to generate evidence of causal association and can go some way to protect against the limitations of observational epidemiology (Davey Smith and Ebrahim, 2004). However, genetic variants identified for alcohol use to date have small effect sizes and might suffer from weak instrument bias, therefore reducing power to detect a true effect. Comparisons using siblings with discordant prenatal alcohol exposure can also be used to disentangle confounding. D'Onofrio and colleagues (2007) sought to investigate the potential causal factors influencing associations between prenatal alcohol exposure and offspring externalizing problems, utilizing sibling controls. The authors found prenatal alcohol exposure to be associated with an increased risk of conduct problems in unrelated offspring. However, when they compared this within siblings, they found offspring who were more exposed to prenatal alcohol did not have greater levels of conduct problems compared to siblings with less prenatal alcohol exposure suggesting the associations shown were likely to be due to other confounding factors related to increased maternal alcohol use. A sibling control design, however, does require a large sample size of siblings with discordant exposure, again causing methodological constraints. Much of the research already conducted on prenatal alcohol exposure using causal inference methods often focuses on moderate to hazardous maternal alcohol use in pregnancy (Lund et al., 2019; Murray et al., 2016), with the findings for light alcohol use and offspring outcomes being less clear. A recent review has shown a lack of studies investigating the causal effect of prenatal alcohol use on offspring, and of those that have there is limited evidence toward either a safe, or detrimental effect of light prenatal alcohol use on offspring outcomes (Mamluk et al., 2017). This highlights the need for further studies using analytical methods that improve causal inference.

Negative control analyses are an alternative method to assess whether associations are due to confounding or likely to be causal. This is done by using exposures or outcomes with similar confounding structures but no plausible biological link (Gage, Munafò and Davey Smith, 2016). If an association is also shown in the negative control analyses, confounding is likely responsible and not the original exposure of interest (Davey Smith, 2008). Comparison of parental exposures on offspring outcomes can be used to test intrauterine effects. For example, both maternal and paternal drinking are likely to be influenced by similar confounding, and therefore, if an association with offspring outcomes is observed, it will be more likely due to intrauterine exposures of alcohol. Negative control analyses have been previously used to investigate the effects of smoking during pregnancy on offspring mental health (Taylor et al., 2017). There are currently few studies using negative control analyses to investigate parental alcohol use during pregnancy, and these studies have mainly focused on offspring externalizing disorders (Eilertsen et al., 2017) or general cognitive ability (Alati et al., 2008). In the current study, we would expect the associations between prenatal alcohol use and offspring depression to be stronger for maternal prenatal alcohol use compared with paternal prenatal alcohol use, demonstrating an intrauterine effect. We sought to investigate associations between both the frequency and pattern of maternal drinking in pregnancy at 18 and 32 weeks gestation and offspring depression, using data from a population‐based longitudinal study. We also investigated whether any associations may reflect a causal effect, using negative control analyses of partner drinking in pregnancy on offspring depression, as both these exposures are likely to be influenced by similar confounding.

## Materials and Methods

### Sample

The Avon Longitudinal Study of Parents and Children (ALSPAC) is an ongoing population‐based study, which recruited pregnant women residing in Avon, UK, with expected dates of delivery between April 1, 1991, and December 31, 1992. The core sample consisted of 14,541 pregnant women, of which 14,062 were live births and alive at 1 year of age. Participants have been regularly followed up through clinic visits and questionnaires. Detailed information about ALSPAC is available on the study website which includes a fully searchable data dictionary of available data (http://www.bris.ac.uk/alspac/researchers/data‐access/data‐dictionary). For further details on the cohort profile, representativeness, and phases of recruitment, see articles by Boyd and colleagues (2013); Fraser and colleagues (2013). Ethics approval for the study was obtained from the ALSPAC Ethics and Law Committee and the Local Research Ethics Committees.

### Measures

#### Exposures

Alcohol consumption during pregnancy was measured by the following: (i) Frequency of drinking: mothers and partners were asked separately the frequency and amount of alcohol consumed (within the past 3 months) at 18 weeks gestation. Participants were advised that 1 glass was equivalent to a pub measure of spirits, half a pint of lager or cider, or a wine glass of wine. Response categories were never, <1 glass per week, 1+ glass per week, 1 to 2 glasses a day, 3 to 9 glasses a day, and ≥ 10 glasses a day. For the analyses, the last 2 categories were combined to 3+ glasses a day due to the very low sample size of the last category (10+ glasses per day: maternal *n* = 8; partner *n* = 26). (ii) Pattern of drinking (binge drinking): mothers and partners were asked the number of days they had drunk the equivalent of 2 pints of beer, 4 glasses of wine, or 4 measures of sprits or more. The definition of binge drinking used here does not necessarily align with certain definitions of binge drinking/binging ((NIAAA), 2019; Stahre et al., 2014). A definition of binge drinking, however, is given to separate it from lower levels of alcohol consumption which does not exceed 1 drink per day. This definition of heavy/binge has been previously used and reported in multiple studies (Alati et al., 2013; Mahedy et al., 2017; Sayal et al., 2009).

Mothers were asked at both 18 weeks (how many times within the first 3 months of pregnancy) and 32 weeks (how many times within the last month) gestation; partners were asked at 18 weeks gestation. Response categories were 0, 1 to 2 days, 3 to 4 days, 5 to 10 days, >10 days, and every day. For our analyses, the last 2 categories were combined to >10 days due to the low sample size of the last category for maternal binge (every day: 18 weeks gestation *n* = 17; 32 weeks gestation *n* = 7), partner binge was derived in the same method to allow comparison against maternal binge (everyday: 18 weeks gestation *n* = 447).

#### Outcomes

Depression in offspring was measured using the computerized version of the Clinical Interview Schedule‐Revised (CIS‐R). The CIS‐R is a computerized interview that is used to diagnose common mental disorders (Lewis et al., 1992), and diagnoses the presence of a depressive episode from the ICD‐10 F32 criteria. The interview can be used to measure both the presence and severity of 14 psychiatric symptoms and diagnoses of other common mental disorders based on ICD‐10 criteria (Head et al., 2013). The current study used the binary measure of a diagnosis of depression to record cases of mild, moderate, or severe depression from the CIS‐R measured at age 18 for the main analyses. Subsequent sensitivity analyses were conducted using the CIS‐R measure at age 24, to investigate whether any associations observed were also present at a later age.

#### Confounders and Sensitivity Analysis

We included characteristics that have previously shown associations with alcohol consumption and offspring psychiatric disorder, and could confound associations (D'Onofrio et al., 2007; Niclasen et al., 2014b; O'Connor and Kasari, 2000; O'Connor and Paley, 2006), while not over adjusting due to the already limited sample size of our cohort. The following indictors of socioeconomic status were included: Mother’s socioeconomic position grouped into 2 categories using the British Registrar General’s Scale (Leete and Fox, 1977) (professional/managerial: i, ii; or other: iii, iv, v) measured during pregnancy, income (divided into quintiles) measured at age 3 and 4 (derived average), homeownership (mortgage/non‐mortgage) measured at 8 weeks gestation, marital status (married or not) measured at 8 weeks gestation, maternal education (university degree, <university degree), sex, parity (firstborn, 2+ born), maternal tobacco (yes/no) and illicit drug use (yes/no) in months 1 to 3 of pregnancy, and maternal depression at 18 weeks gestation (scores >12 highly associated with a diagnosis of depression) measured by the Edinburgh Postnatal Depression Scale (EPDS) (Cox et al., 1996). Main effects were further adjusted for maternal polygenic risk scores (PRS) for depression to investigate possible shared genetic effects. The maternal PRS for depression was calculated using single nucleotide polymorphisms (SNPs) for depression identified in a genome‐wide association study of major depressive disorder (MDD) (Wray et al., 2018).

### Statistical Analyses

We used logistic regression to investigate associations between maternal and partner alcohol frequency (18 weeks gestation), binge drinking (18 and 32 weeks gestation), and a diagnosis of depression (CIS‐R) at age 18. Comparisons were made between the never drank controls in each alcohol exposure group and each alcohol frequency/pattern group. Analyses were conducted using Stata version 14.2, StataCorp. 2015. Stata Statistical Software: Release 14. College Station, TX: StataCorp LP.

The impact of confounders on these associations was explored by comparing unadjusted estimates, to those adjusted for socioeconomic variables (adjusted model 1) and those further adjusted for maternal behavior during pregnancy (e.g., other drug use during pregnancy/maternal depression) variables (adjusted model 2), and for partner alcohol use (frequency or pattern, dependant on exposure) during pregnancy (18 weeks gestation only) (adjusted model 3). By increasing, the number of items adjusted for the sample size decreased, as individuals with missing data are excluded from analysis. Therefore, as a sensitivity analysis, all analyses were conducted using the full sample and then repeated only on participants with complete data.

Postestimation tests were conducted using seemingly unrelated estimation *(suest)* tests, with bootstrapping (*n* = 1,000). These analyses calculated the difference in associations between maternal and partner prenatal alcohol use and offspring depression at age 18, and the ratio of odds ratios was used to indicate the difference. This was conducted separately in both an unadjusted model and a mutually adjusted model including partners’ (maternal and partner) alcohol consumption.

Missing data were present within all variables used in our analyses (see Table S16); we therefore used multiple imputation (MI) to reduce bias. Missing data for each variable ranged from 12% to 70% within the measures used; however, multiple imputation has been shown to be valid in reducing bias even when large amounts of missing data are present as long as suitable auxiliary measures can be applied to the imputation model (Madley‐Dowd et al., 2019). Multiple imputation by chained equation (MICE) in Stata (Royston and White, 2011) was used to generate a maximum dataset comprising of 100 imputed datasets, each with 10 cycles. Generation of more than 1 imputation model allowed for the uncertainty in predicting missing data, by adding variability to the imputed values in each dataset, which are then averaged together. The variability in results between each dataset reflects the uncertainty associated with the missing values, and using Rubin’s rules, standard errors are calculated which account for the variability in these results (Sterne et al., 2009). By averaging the distribution of the missing data from the observed data, valid assumptions can be made which account for variability. This method assumes any systematic differences between the missing and observed values can be explained by differences in observed data and are missing at random (Sterne et al., 2009). Multiple auxiliary variables available from the ALSPAC cohort were used to assist in the imputation. These included the predictive factors used in the main analysis (e.g., socioeconomic position), as well as other measures related to the outcomes (e.g., EPDS), and earlier offspring depressive measures such Mood and Feelings Questionnaire (MFQ) (Angold et al., 1995).

## Results

Overall, 16% of mothers (2,088 of 13,195) reported drinking at least 1 alcoholic drink per week in the first 3 months of pregnancy. At 18 weeks gestation 17% of mothers (2,234 of 13,149) reported binge drinking for at least 1 to 2 days within the past month. For mothers who provided information on alcohol frequency or pattern of drinking, 4,191 and 4,169 offspring, respectively, provided information for CIS‐R diagnosis of depression at age 18. Mother and offspring characteristics for full sample analysis are presented in Tables 1 and 2. All further presented results are for imputed analyses unless stated otherwise.

**Table 1:** Mother and Offspring Socioeconomic Factors, by Pattern of Drinking (Number of Times 4+ Units of Alcohol in Past Month at 18 Weeks Gestation)

	Drinking category				
	None	1 to 2 days	3 to 4 days	5 to 10 days	>10 days
Socioeconomic position					
i–ii	3,212	276	116	52	59
iii–v	5,071	595	247	130	118
Homeownership					
Homeowner	7,975	754	313	158	167
Nonhomeowner	2,547	378	146	99	110
Marital status					
Married	8,213	753	296	164	163
Not married	2,363	379	161	92	113
Maternal education					
University degree	1,440	84	23	19	19
No university degree	8,603	980	415	221	239
Offspring gender					
Male	5,612	629	234	137	153
Female	5,248	553	245	129	135
Parity					
Firstborn	4,922	474	178	109	103
2^nd^ + born	5,812	694	291	158	182
Smoked during pregnancy					
No	8,496	740	267	149	166
Yes	2,419	452	215	118	127
Drug use during pregnancy					
No	10,748	1,168	464	255	284
Yes	33	7	11	7	5
Depression 18 weeks gestation					
No	8,744	894	334	188	198
Yes	1,260	195	91	50	63

i–ii: Professional and managerial occupations. iii–v: Nonmanual/manual/semi‐skilled manual and unskilled manual.

**Table 2:** Sample Size for Exposure Measures of Frequency and Pattern of Alcohol Consumption for Mother and Partners at 18 and 32 Weeks Gestation

Frequency	None *n* *(%)*	<1 glass per week *n* *(%)*	1 + glass per week *n* *(%)*	1 to 2 glasses per day *n* *(%)*	3+ glasses per day *n* *(%)*
Mother 18 weeks gestation	6,002 (45%)	5,105 (39%)	1,834 (14%)	212 (2%)	42 (<1%)
Partner 18 weeks gestation	488 (5%)	2,408 (24%)	4,941 (50%)	1,521 (15%)	483 (5%)

Pattern	None *n* *(%)*	1 to 2 days *n* *(%)*	3 to 4 days *n* *(%)*	5 to 10 days *n* *(%)*	>10 days *n* *(%)*
Mother 18 weeks gestation	10,915 (82%)	1,192 (9%)	482 (4%)	267 (2%)	293 (2%)
Mother 32 weeks gestation	7,255 (83%)	877 (10%)	353 (4%)	179 (2%)	116 (1%)
Partner 32 weeks gestation	1,846 (19%)	1,763 (18%)	1,904 (19%)	2,513 (25%)	1,915 (19%)

### Maternal Alcohol Consumption and Offspring Depression

Individuals whose mothers consumed any alcohol at 18 weeks gestation were at increased odds of having a diagnosis of depression at age 18 (unadjusted OR = 1.18, 95% CI 1.03 to 1.34). After adjustment for socioeconomic and maternal behaviors, these associations were attenuated only slightly (OR = 1.13, 95% CI 0.99 to 1.29, Table 3). Further adjustment for partner alcohol use strengthened the association slightly (OR = 1.17, 95% CI 1.02 to 1.34).

**Table 3:** How Often Mothers and Partners Consumed Alcoholic Drinks at 18 Weeks Gestation and Offspring Depression Age 18

	*n* = 13,480	Unadjusted		Adjusted^a^		Adjusted^b^		Adjusted^c^	
		OR (CI)	*p*	OR (CI)	*p*	OR (CI)	*p*	OR (CI)	*p*
Mothers	Never	1.00 (ref)	0.036^d^	1.00 (ref)	0.087^d^	1.00 (ref)	0.237^d^	1.00 (ref)	0.129^d^
	<1 glass per week	1.09 (0.88 to 1.36)		1.11 (0.89 to 1.39)		1.09 (0.87 to 1.37)		1.12 (0.90 to 1.41)	
	1+ glass per week	1.90 (0.88 to 1.60)		1.17 (0.86 to 1.60)		1.11 (0.81 to 1.52)		1.19 (0.87 to 1.63)	
	1 to 2 glasses per day	1.93 (0.92 to 4.03)		1.81 (0.84 to 3.87)		1.59 (0.73 to 3.46)		1.85 (0.83 to 4.11)	
	3+ glasses per day	5.34 (1.29 to 22.01)		4.91 (1.07 to 22.44)		4.06 (0.86 to 19.31)		4.45 (0.92 to 21.46)	
	Linear trend	1.18 (1.03 to 1.34)	0.015	1.17 (1.02 to 1.33)	0.024	1.13 (0.99 to 1.29)	0.075	1.17 (1.02 to 1.34)	0.025
Fathers	Never	1.00 (ref)	0.087^d^	1.00 (ref)	0.203^d^	1.00 (ref)	0.209^d^	1.00 (ref)	0.108^d^
	<1 glass per week	1.21 (0.69 to 2.12)		1.27 (0.71 to 2.26)		1.28 (0.71 to 2.29)		1.24 (0.69 to 2.22)	
	1 + glass per week	0.88 (0.48 to 1.60)		0.96 (0.51 to 1.78)		0.97 (0.51 to 1.81)		0.90 (0.48 to 1.71)	
	1 to 2 glasses per day	0.77 (0.41 to 1.45)		0.85 (0.44 to 1.65)		0.86 (0.44 to 1.67)		0.77 (0.39 to 1.53)	
	3 + glasses per day	0.97 (0.45 to 2.08)		0.99 (0.45 to 2.18)		0.97 (0.44 to 2.16)		0.85 (0.38 to 1.93)	
	Linear trend	0.88 (0.77 to 1.02)	0.086	0.90 (0.77 to 1.04)	0.161	0.90 (0.77 to 1.04)	0.154	0.87 (0.74 to 1.01)	0.071

^a^ Adjusted for: socioeconomic position, income, homeownership, marital status, maternal education, gender, parity.

^b^ Adjusted for: socioeconomic position, income, homeownership, marital status, maternal education, gender, parity, maternal tobacco use during 1 to 3 months of pregnancy, maternal illicit drug use during 1 to 3 months of pregnancy, maternal depression 18 weeks gestation.

^c^ Adjusted for: socioeconomic position, income, homeownership, marital status, maternal education, gender, parity, maternal tobacco use during 1 to 3 months of pregnancy, maternal illicit drug use during 1 to 3 months of pregnancy, maternal depression 18 weeks gestation, how often partner consumed alcohol at 18 weeks gestation.

^d^ Wald test.

There was no clear evidence that the number of days when ≥4 alcoholic drinks were consumed over the last month at 18 or 32 weeks gestation was associated with offspring depression at age 18 (Tables S1 and S12).

In sensitivity analyses of offspring depression at age 24, we observed similar (although slightly weaker) results as for offspring depression at age 18, for mothers who consumed any alcohol at 18 weeks gestation (unadjusted OR = 1.07, 95% CI 0.93 to 1.24). Individuals whose mothers consumed ≥4 alcoholic drinks over the last month at 32 weeks gestation were at increased odds of having a diagnosis of depression at age 24 (unadjusted OR = 1.28, 95% CI 1.12 to 1.47). After adjustment for socioeconomic and maternal behaviors, these associations were attenuated only slightly (OR = 1.20, 95% CI 1.03 to 1.40, see Table S15).

The findings from the full sample and complete case analyses did not differ substantially from the imputed analyses (see Tables S2–S4 and S7–S9).

### Partner Alcohol Consumption and Offspring Depression

Paternal alcohol use at 18 weeks gestation showed no clear evidence of association with offspring depression at age 18, for both frequency (unadjusted OR = 0.88, 95% CI 0.77 to 1.02, Table 3) and pattern of alcohol use (unadjusted OR = 0.95, 95% CI 0.94 to 1.19, Table S12).

In sensitivity analyses of offspring depression at age 24, we found no clear evidence that offspring of mothers whose partners consumed any alcohol at 18 weeks gestation were at increased odds of having a diagnosis of depression at age 24 (Table S13). There was also no clear evidence that the number of days when ≥4 alcoholic drinks were consumed over the last month at 18 weeks gestation by partners was associated with offspring depression at age 24 (Table S14).

The findings from the full sample and complete case analyses did not differ substantially from the imputed analyses (see Tables S5 and S6, S10 and S11).

Exploratory analyses were conducted to investigate whether the associations shown between maternal alcohol consumption during pregnancy and offspring depression were due to shared genetic risk for depression. The associations previously shown between frequency of alcohol consumed during pregnancy and offspring depression at age 18 were slightly attenuated after further adjustment for maternal PRS for depression (OR = 1.09, 95% CI 0.88 to 1.35).

### Differences Between Maternal and Partner Alcohol Exposure

Postestimation tests indicated a positive difference between associations of maternal and partner alcohol consumption at 18 weeks gestation and offspring depression at age 18 (i.e., stronger association for maternal alcohol exposure compared with partner alcohol use), both in unadjusted models (OR 1.36, CI 1.09 to 1.68) and models mutually adjusted for partner (maternal and partner) alcohol use (OR 1.41, CI 1.07 to 1.84) (Table 4).

**Table 4:** Differences Between Associations of Maternal and Partner Alcohol Consumption at 18 Weeks Gestation and Offspring Depression at Age 18

	OR	CI	*p* Value
Unadjusted	1.36	1.09 to 1.68	0.005
Mutually adjusted	1.41	1.07 to 1.84	0.013

^a^ Ratio of OR of maternal to partner alcohol consumption and offspring depression.

## Discussion

We investigated the association between maternal alcohol consumption in pregnancy (frequency and pattern) and offspring depression in a population‐based study. Our results suggest that the amount of alcohol mothers consumed during pregnancy at 18 weeks gestation is associated with offspring depression at age 18 and indicate a linear trend between amount of alcohol mothers drank in pregnancy and offspring risk of depression. For partner alcohol consumption, there was no clear evidence of association of either the amount of alcohol consumed, or drinking ≥4 alcoholic drinks with offspring depression; indeed, the point estimates shown for the negative control analyses suggested associations in the opposite direction for maternal and partner drinking on offspring depression. Furthermore, postestimation tests found a positive difference between the association of maternal and partner alcohol use on offspring depression, showing a stronger association for maternal compared with partner alcohol use in both unadjusted and mutually adjusted models. These findings suggest that the associations shown for maternal alcohol consumption in pregnancy and offspring depression may be causal, and these associations are unlikely to be due to the result of shared confounding structures between maternal and paternal exposures. Such findings have implications for women trying to conceive who could be unaware of a successful pregnancy in the early stages of gestation while still consuming alcohol (Floyd, Decouflé and Hungerford, 1999). Our findings therefore support abstinence from alcohol for women who are trying to conceive.

Of the research previously conducted on PAE and offspring internalizing disorders, there have been unclear findings. A recent review found limited evidence of an effect on offspring internalizing outcomes (Mamluk et al., 2017); however, there were only 2 studies identified within this review which investigated the effect on internalizing disorders. Light alcohol use has also been shown to be protective, with increased PAE being associated with a decrease in offspring internalizing problems (Kelly et al., 2012; Robinson et al., 2010a). However, the authors note that this finding may be due to the over representation of socially disadvantaged families included in the analysis, and therefore, any associations found may have been driven by residual confounding. A review by Kelly and colleagues highlighted the changes in social behavior that can occur with alcohol consumption, and suggested that offspring who may be exposed to alcohol prenatally may have differences in the way their social behavior was grounded in early life (Kelly, Day and Streissguth, 2000). Associations found could therefore still be due to residual confounding which may have influenced early life behaviors.

Our use of a negative control comparison of paternal drinking in pregnancy provides some support for the possibility that the observations we have observed may be causal. By using a long follow‐up for the main outcome (at age 18), our findings suggest that any associations shown within the offspring are likely to persist throughout childhood and into adulthood. Although we also found that the associations were attenuated for amount of alcohol consumed when measuring prenatal alcohol use against offspring depression at a later timepoint (age 24), the direction of association remained the same.

Although the associations we observed are relatively weak, they may nevertheless be important at a population level, particularly as depression is a common mental health disorder affecting more than 300 million people globally (WHO, 2017). The population‐attributable fraction (PAF) was calculated for alcohol exposure using UK prevalence of alcohol use during pregnancy from a recent meta‐analysis (O'Keeffe et al., 2015) for the comparison numerators and denominators. The PAF for the contribution of maternal alcohol use in pregnancy on offspring depression ranged between 0.05 to 0.15. This suggests that if the associations observed were causal and precisely estimated, the percentage of depression cases that are preventable by almost removing alcohol consumption during pregnancy ranges between 5% and 15%, depending on which estimate this is based on. Our findings therefore provide support for guidelines recommending complete abstinence from alcohol during pregnancy, or for women trying to conceive.

An advantage of the ALSPAC cohort, where recruitment occurred during 1990 to 1991, is that attitudes in the UK toward drinking in pregnancy were likely to have been different to current day with less stigma associated with drinking in pregnancy, meaning that mothers may have been more likely to truthfully report alcohol consumption. However, underreporting of alcohol use may still have occurred if mothers were not aware they were pregnant until later stages of pregnancy, therefore misrepresenting the true level of alcohol exposure as the measures relied on valid self‐report. If alcohol use was underreported, the findings we observed are likely to be more conservative and a larger association could have been shown if there was a more biologically valid way to assess maternal alcohol consumption.

There are limitations that should be considered when interpreting these results. Firstly, there is sample attrition from enrollment to the outcome measurement at age 18. Characteristics between responders and nonresponders in the ALSPAC study could cause selection bias. However, as the complete case analyses and those using the imputed dataset do not differ substantially, selection bias is unlikely to have affected the reported associations. Previous studies investigating biases within the ALSPAC cohort have found the strength of associations to not be greatly affected by selection bias and sample attrition (Wolke et al., 2009). Secondly, the associations may also be due to a shared genetic risk for depression, which is expressed as different phenotypes in mothers and offspring. However, further analyses which adjusted for maternal PRS for depression, found the associations shown previously for maternal alcohol use during pregnancy and offspring depression were attenuated only slightly. This suggests that a shared genetic risk for depression from maternal genetic risk may only partially account for offspring depression in our study.

Our study highlights the potentially long‐lasting detrimental effects of maternal alcohol consumption in pregnancy on offspring mental health. Although the associations we observed are small, they may nevertheless be important at a population level. The negative control comparison of paternal alcohol use during pregnancy provides some evidence that the associations found may be causal. However, further research is needed to determine with greater confidence whether these associations are indeed causal, and the result of intrauterine exposure. This may require the use of other methodological approaches, such as Mendelian randomization, and sibling comparisons with offspring discordant for maternal alcohol consumption in pregnancy.

## Conflicts of Interest

The authors have no conflicts of interest to declare.

## Author Contributions

K.E.E, N.J.T., and M.R.M. conceived the study. K.E.E. conducted the analysis and drafted the initial manuscript. All authors assisted with interpretation of results, commented on multiple drafts of the manuscript and approved the final version for publication.

## Research Ethics

Ethics approval for the ALSPAC cohort study was obtained from the ALSPAC Ethics and Law Committee and the Local Research Ethics Committees. Informed consent was obtained from all individual participants included in the study.

## Disclaimer

This publication is the work of all authors, and Kayleigh Easey, Nicholas Timpson, and Marcus Munafò will serve as guarantors for the contents of this study.

## Supporting information


**Table S1**. Depression at age 18 and maternal alcohol binge at 32 weeks gestation, imputed data.
**Table S2** **.** Depression at age 18 and maternal alcohol amount at 18 weeks gestation, full sample.
**Table S3** **.** Depression at age 18 and maternal alcohol binge at 18 weeks gestation, full sample.
**Table S4** **.** Depression at age 18 and maternal alcohol binge at 32 weeks gestation, full sample.
**Table S5** **.** Depression at age 18 and partners alcohol amount at 18 weeks gestation, full sample.
**Table S6** **.** Depression at age 18 and partners alcohol binge at 18 weeks gestation, full sample.
**Table S7** **.** Depression at age 18 and maternal alcohol amount at 18 weeks gestation, complete case.
**Table S8** **.** Depression at age 18 and maternal alcohol binge at 18 weeks gestation, complete case.
**Table S9** **.** Depression at age 18 and maternal alcohol binge at 32 weeks gestation, complete case.
**Table S10** **.** Depression at age 18 and partners alcohol amount at 18 weeks gestation, complete case.
**Table S11** **.** Depression at age 18 and partners alcohol binge at 18 weeks gestation, complete case.
**Table S12** **.** Depression at age 18 and mother and partner alcohol binge at 18 weeks gestation, imputed data.
**Table S13** **.** Depression at age 24 and mother and partner alcohol amount at 18 weeks gestation, imputed data.
**Table S14** **.** Depression at age 24 and mother and partner alcohol binge at 18 weeks gestation, imputed data.
**Table S15** **.** Depression age 24 and maternal alcohol binge 32 weeks gestation imputed data.
**Table S16** **.** Proportions of missing data for exposures, outcomes and confounders.

## Data Availability

ALSPAC data used for this submission are available upon application to the Executive of ALSPAC (alspac-exec@bristol.ac.uk). The ALSPAC data management plan (http://www.bristol.ac.uk/alspac/researchers/dataaccess/documents/alspac‐data‐management‐plan.pdf) describes in detail the policy regarding data sharing, which is through a system of managed open access.
