# Supplementary material for: Association of Prenatal Alcohol Exposure and Offspring Depression: A Negative Control Analysis of Maternal and Partner Consumption
Source: Alcohol Clin Exp Res. 2020 Apr 21;44(5):1132–40. doi: 10.1111/acer.14324 (PMC7341445; doi:10.1111/acer.14324)
Supplement: Supplementary file 1 — Table S1. Depression at age 18 and maternal alcohol binge at 32 weeks gestation, imputed data. Table S2 . Depression at age 18 and maternal alcohol amount at 18 weeks gestation, full sample. Table S3 . Depression at age 18 and maternal alcohol binge at 18 weeks gestation, full sample. Table S4 . Depression at age 18 and maternal alcohol binge at 32 weeks gestation, full sample. Table S5 . Depression at age 18 and partners alcohol amount at 18 weeks gestation, full sample. Table S6 . Depression at age 18 and partners alcohol binge at 18 weeks gestation, full sample. Table S7 . Depression at age 18 and maternal alcohol amount at 18 weeks gestation, complete case. Table S8 . Depression at age 18 and maternal alcohol binge at 18 weeks gestation, complete case. Table S9 . Depression at age 18 and maternal alcohol binge at 32 weeks gestation, complete case. Table S10 . Depression at age 18 and partners alcohol amount at 18 weeks gestation, complete case. Table S11 . Depression at age 18 and partners alcohol binge at 18 weeks gestation, complete case. Table S12 . Depression at age 18 and mother and partner alcohol binge at 18 weeks gestation, imputed data. Table S13 . Depression at age 24 and mother and partner alcohol amount at 18 weeks gestation, imputed data. Table S14 . Depression at age 24 and mother and partner alcohol binge at 18 weeks gestation, imputed data. Table S15 . Depression age 24 and maternal alcohol binge 32 weeks gestation imputed data. Table S16 . Proportions of missing data for exposures, outcomes and confounders. [file ACER-44-1132-s001.docx]

**Supplementary Material**

**Supplementary Table 1: Depression at age 18 and maternal alcohol binge at 32 weeks gestation, imputed data**

|  | Unadjusted | | Adjusted^1^ | | Adjusted^2^ | |
| --- | --- | --- | --- | --- | --- | --- |
| *n* = 13480 | OR (CI) | *P* | OR (CI) | *P* | OR (CI) | *P* |
| None | 1.00 (ref) | 0.564^a^ | 1.00 (ref) | 0.735^a^ | 1.00 (ref) | 0.724^a^ |
| 1-2 days | 1.09 (0.77-1.55) |  | 1.01 (0.70-1.45) |  | 0.96 (0.66-1.39) |  |
| 3-4 days | 1.50 (0.93-2.42) |  | 1.38 (0.84-2.27) |  | 1.27 (0.76-2.12) |  |
| 5-10 days | 0.86 (0.35-2.14) |  | 0.80 (0.31-2.03) |  | 0.69 (0.26-1.81) |  |
| >10 days | 0.90 (0.32-2.57) |  | 0.81 (0.28-2.36) |  | 0.69 (0.23-2.03) |  |
| Linear trend | 1.05 (0.91-1.22) | 0.491 | 1.02 (0.87-1.19) | 0.846 | 0.97 (0.83-1.15) | 0.742 |
| Model^1^ adjusted initially for socioeconomic position, income, home ownership, marital status, maternal education, gender and parity. Model^2^ further adjusted for tobacco use during 1-3 months of pregnancy, illicit drug use during 1-3 months of pregnancy and maternal depression at 18 weeks gestation  ^a^Wald test | | | | | | |

**Supplementary Table 2: Depression at age 18 and maternal alcohol amount at 18 weeks gestation, full sample**

|  | Unadjusted *n* = 4191 |  | Adjusted^1^ *n* = 3203 | | |  | Adjusted^2^ *n* = 3027 | |  | Adjusted^3^ *n* = 2566 | |
| --- | --- | --- | --- | --- | --- | --- | --- | --- | --- | --- | --- |
|  | OR (CI) | *P* | OR (CI) | | | *P* | OR (CI) | | *P* | OR (CI) | *P* |
| Never | 1.00 (ref) | 0.060^b^ | 1.00 (ref) |  |  | 0.640^b^ | 1.00 (ref) |  | 0.742^b^ | 1.00 (ref) | 0.734^b^ |
| <1 glass per week | 1.16 (0.91-1.49) |  | 1.01 (0.76-1.36) | | |  | 0.92 (0.68-1.25) | |  | 0.89 (0.64-1.25) |  |
| 1+ glass per week | 1.12 (0.79-1.60) |  | 1.09 (0.73-1.65) | | |  | 0.97 (0.63-1.49) | |  | 0.96 (0.59-1.56) |  |
| 1-2 glasses per day | 1.86 (0.87-3.98) |  | 1.57 (0.64-3.86) | | |  | 1.45 (0.57-3.67) | |  | 1.82 (0.56-5.86) |  |
| 3+ glasses per day | 5.78 (1.76-19.02) |  | 3.21(0.63-16.44) | | |  | 2.33 (0.44-12.49) | |  | 2.19 (0.23-20.84) |  |
| Linear trend | 1.16 (1.01-1.34) | 0.035 | 1.09 (0.92-1.30) | | | 0.297 | 1.03 (0.86-1.23) | | 0.742 | 1.01 (0.82-1.25) | 0.895 |

Model^1^ adjusted initially for socioeconomic position, income, home ownership, marital status, maternal education, gender and parity.

Model^2^ further adjusted for tobacco use during 1-3 months of pregnancy, illicit drug use during 1-3 months of pregnancy and maternal depression at 18 weeks gestation.

Model^3^ further adjusted for partner alcohol consumption during pregnancy.

^b^Omnibus p-value

**Supplementary Table 3: Depression at age 18 and maternal alcohol binge at 18 weeks gestation, full sample**

|  | Unadjusted *n =* 4169 | | | Adjusted^1^ *n =* 3196 | | | Adjusted^2^ *n =* 3021 | | Adjusted^3^ *n =* 2580 | |
| --- | --- | --- | --- | --- | --- | --- | --- | --- | --- | --- |
|  | OR (CI) | | *P* | OR (CI) | | *P* | OR (CI) | *P* | OR (CI) | *P* |
| none | 1.00 (ref) |  | 0.123^b^ | 1.00 (ref) |  | 0.161^b^ | 1.00 (ref) | 0.340^b^ | 1.00 (ref) | 0.352^b^ |
| 1-2 days | 1.35 (0.92-2.00) | |  | 1.36 (0.86-2.14) | |  | 1.16 (0.71-1.90) |  | 1.29 (0.76-2.20) |  |
| 3-4 days | 1.88 (1.11-3.18) | |  | 1.81 (0.96-3.42) | |  | 1.64 (0.83-3.25) |  | 1.58 (0.74-3.37) |  |
| 5-10 days | 1.29 (0.55-3.02) | |  | 1.52 (0.63-3.67) | |  | 1.39 (0.57-3.38) |  | 1.72 (0.68-4.35) |  |
| >10 days | 1.39 (0.63-3.06) | |  | 0.47 (0.11-1.96) | |  | 0.41 (0.09-1.81) |  | 0.37 (0.49-2.82) |  |
| Linear trend | 1.16 (1.02-1.32) | | 0.026 | 1.08 (0.91-1.27) | | 0.373 | 1.03 (0.86-1.23) | 0.750 | 1.08 (0.89-1.32) | 0.435 |

Model^1^ adjusted initially for socioeconomic position, income, home ownership, marital status, maternal education, gender and parity. Model^2^ further adjusted for tobacco use during 1-3 months of pregnancy, illicit drug use during 1-3 months of pregnancy and maternal depression at 18 weeks gestation. Model^3^ further adjusted for partner alcohol consumption during pregnancy.

^b^Omnibus p-value

**Supplementary Table 4: Depression at age 18 and maternal alcohol binge at 32 weeks gestation, full sample**

|  | Unadjusted *n=* 2932 | | Adjusted^1^ *n=*2316 | | Adjusted^2^ *n=* 2213 | |
| --- | --- | --- | --- | --- | --- | --- |
|  | OR (CI) | *P* | OR (CI) | *P* | OR (CI) | *P* |
| none | 1.00 (ref) | 0.039^b^ | 1.00 | 0.115^b^ | 1.00 (ref) | 0.426^b^ |
| 1-2 days | 1.07 (0.66-1.73) |  | 0.95 (0.54-1.66) |  | 0.83 (0.45-1.51) |  |
| 3-4 days | 2.68 (1.47-4.85) |  | 2.32 (1.16-4.65) |  | 1.73 (0.81-3.74) |  |
| 5-10 days | 0.52 (0.13-2.17) |  | 0.58 (0.14-2.44) |  | 0.56 (0.13-2.40) |  |
| >10 days | 1.48 (0.44-4.93) |  | 2.62 (0.72-9.49) |  | 1.84 (0.49-6.96) |  |
| Linear trend | 1.14 (0.95-1.36) | 0.150 | 1.16 (0.94-1.42) | 0.172 | 1.06 (0.84-1.32) | 0.628 |

Model^1^ adjusted initially for socioeconomic position, income, home ownership, marital status, maternal education, gender and parity.

Model^2^ further adjusted for tobacco use during 1-3 months of pregnancy, illicit drug use during 1-3 months of pregnancy and maternal depression at 18 weeks gestation.

^b^Omnibus p-value

**Supplementary Table 5: Depression at age 18 and partners alcohol amount at 18 weeks gestation, full sample**

|  | Unadjusted *n* = 3416 | | | Adjusted^1^ *n* = 2708 | | Adjusted^2^ *n* = 2572 | | Adjusted^3^ *n* = 2566 | |
| --- | --- | --- | --- | --- | --- | --- | --- | --- | --- |
|  | OR (CI) | | *P* | OR (CI) | *P* | OR (CI) | *P* | OR (CI) | *P* |
| Never | | 1.00 (ref) | 0.057^b^ | 1.00 (ref) | 0.398^b^ | 1.00 (ref) | 0.338^b^ | 1.00 (ref) | 0.339^b^ |
| <1 glass per week | | 1.41 (0.69-2.90) |  | 1.42 (0.59-3.43) |  | 1.63 (0.62-4.27) |  | 1.64 (0.62-4.30) |  |
| 1+ glass per week | | 0.96 (0.48-1.94) |  | 1.06 (0.45-2.53) |  | 1.27 (0.49-3.27) |  | 1.26 (0.49-3.26) |  |
| 1-2 glasses per day | | 0.80 (0.37-1.71) |  | 0.91 (0.36-2.31) |  | 0.97 (0.35-2.68) |  | 0.95 (0.34-2.67) |  |
| 3+ glasses per day | | 1.10 (0.45-2.65) |  | 1.24 (0.43-3.55) |  | 1.24 (0.39-3.89) |  | 1.20 (0.38-3.82) |  |
| Linear trend | | 0.86 (0.74-1.00) | 0.045 | 0.90 (0.75-1.07) | 0.245 | 0.88 (0.73-1.05) | 0.163 | 0.88 (0.73-1.06) | 0.172 |

Model^1^ adjusted initially for socioeconomic position, income, home ownership, marital status, maternal education, gender and parity.

Model^2^ further adjusted for tobacco use during 1-3 months of pregnancy, illicit drug use during 1-3 months of pregnancy and maternal depression at 18 weeks gestation.

Model^3^ further adjusted for partner alcohol consumption during pregnancy.

^b^Omnibus p-value

**Supplementary Table 6: Depression at age 18 and partners alcohol binge at 18 weeks gestation, full sample**

|  | Unadjusted *n* = 3440 | | Adjusted^1^ *n* = 2723 | | Adjusted^2^ *n* = 2586 | | Adjusted^3^ *n* = 2580 | |
| --- | --- | --- | --- | --- | --- | --- | --- | --- |
|  | OR (CI) | *P* | OR (CI) | *P* | OR (CI) | *P* | OR (CI) | *P* |
| None | 1.00 (ref) | 0.657^b^ | 1.00 (ref) | 0.329^b^ | 1.00 (ref) | 0.304^b^ | 1.00 (ref) | 0.289^b^ |
| 1-2 days | 1.01 (0.67-1.53) |  | 1.06 (0.65-1.74) |  | 1.04 (0.63-1.76) |  | 1.04 (0.62-1.75) |  |
| 3-4 days | 0.87 (0.57-1.32) |  | 0.97 (0.59-1.58) |  | 0.98 (0.59-1.62) |  | 0.97 (0.58-1.61) |  |
| 5-10 days | 1.06 (0.73-1.54) |  | 1.27 (0.81-1.99) |  | 1.19 (0.75-1.89) |  | 1.17 (0.73-1.86) |  |
| >10 days | 0.82 (0.53-1.25) |  | 0.80 (0.47-1.33) |  | 0.71 (0.42-1.22) |  | 0.69 (0.40-1.20) |  |
| Linear trend | 0.97 (0.89-1.07) | 0.544 | 0.99 (0.89-1.10) | 0.797 | 0.96 ((0.86-1.07) | 0.465 | 0.95 (0.85-1.07) | 0.413 |

Model^1^ adjusted initially for socioeconomic position, income, home ownership, marital status, maternal education, gender and parity.

Model^2^ further adjusted for tobacco use during 1-3 months of pregnancy, illicit drug use during 1-3 months of pregnancy and maternal depression at 18 weeks gestation.

Model^3^ further adjusted for partner alcohol consumption during pregnancy.

^b^Omnibus p-value

| **Supplementary Table 7: Depression at age 18 and maternal alcohol amount at 18 weeks gestation, complete case** | | | | | | |
| --- | --- | --- | --- | --- | --- | --- |
|  | Unadjusted | | Adjusted^1^ | | Adjusted^2^ | |
| *n* = 3027 | OR (CI) | *P* | OR (CI) | *P* | OR (CI) | *P* |
| Never | 1.00 (ref) | 0.367^b^ | 1.00 (ref) | 0.554^b^ | 1.00 (ref) | 0.742^b^ |
| <1 glass per week | 0.99 (0.73-1.33) |  | 0.97 (0.71-1.31) |  | 0.92 (0.68-1.25) |  |
| 1+ glass per week | 1.11 (0.74-1.69) |  | 1.07 (0.70-1.64) |  | 0.97 (0.63-1.49) |  |
| 1-2 glasses per day | 2.01 (0.83-4.87) |  | 1.71 (0.69-4.27) |  | 1.45 (0.57-3.67) |  |
| 3+ glasses per day | 3.63 (0.74-17.72) |  | 3.17 (0.61-16.35) |  | 2.33 (0.44-12.49) |  |
| Linear trend | 1.12 (0.94-1.33) | 0.196 | 1.09 (0.91-1.30) | 0.340 | 1.03 (0.86-1.23) | 0.742 |

Model^1^ adjusted initially for socioeconomic position, income, home ownership, marital status, maternal education, gender and parity.

Model^2^ further adjusted for tobacco use during 1-3 months of pregnancy, illicit drug use during 1-3 months of pregnancy and maternal depression at 18 weeks gestation.

^b^Omnibus p-value

| **Supplementary Table 8: Depression at age 18 and maternal alcohol binge at 18 weeks gestation, complete case** | | | | | | | | |
| --- | --- | --- | --- | --- | --- | --- | --- | --- |
|  | Unadjusted | | Adjusted^1^ | | | Adjusted^2^ | | |
| *n* = 3021 | OR (CI) | *P* | OR (CI) | | *P* | OR (CI) | | *P* |
| none | 1.00 (ref) | 0.118^b^ | 1.00 (ref) |  | 0.256^b^ | 1.00 (ref) |  | 0.340^b^ |
| 1-2 days | 1.38 (0.86-2.21) |  | 1.24 (0.76-2.01) | |  | 1.16 (0.71-1.90) | |  |
| 3-4 days | 2.09 (1.09-4.02) |  | 1.90 (0.97-3.70) | |  | 1.64 (0.83-3.25) | |  |
| 5-10 days | 1.79 (0.75-4.25) |  | 1.51 (0.62-3.66) | |  | 1.39 (0.57-3.38) | |  |
| >10 days | 0.64 (0.15-2.67) |  | 0.53 (0.13-2.26) | |  | 0.41 (0.09-1.81) | |  |
| Linear trend | 1.14 (0.97-1.35) | 0.111 | 1.08 (0.91-1.29) | | 0.354 | 1.03 (0.86-1.23) | | 0.750 |

Model^1^ adjusted initially for socioeconomic position, income, home ownership, marital status, maternal education, gender and parity.

Model^2^ further adjusted for tobacco use during 1-3 months of pregnancy, illicit drug use during 1-3 months of pregnancy and maternal depression at 18 weeks gestation.

^b^Omnibus p-value

**Supplementary Table 9: Depression at age 18 and maternal alcohol binge at 32 weeks gestation, complete case**

|  | Unadjusted |  | Adjusted^1^ |  | Adjusted^2^ |  |
| --- | --- | --- | --- | --- | --- | --- |
| *n* = 2213 | OR (CI) | *P* | OR (CI) | *P* | OR (CI) | *P* |
| 0. none | 1.00 (ref) | 0.139^b^ | 1.00 (ref) | 0.298^b^ | 1.00 (ref) | 0.426^b^ |
| 1. 1-2 days | 1.06 (0.60-1.88) |  | 0.95 (0.53-1.70) |  | 0.83 (0.45-1.51) |  |
| 2. 3-4 days | 2.47 (1.19-5.13) |  | 1.96 (0.92-4.16) |  | 1.73 (0.81-3.74) |  |
| 3. 5-10 days | 0.67 (0.16-2.80) |  | 0.63 (0.15-2.67) |  | 0.56 (0.13-2.40) |  |
| 4. >10 days | 2.57 (0.74-8.93) |  | 2.53 (0.70-9.23) |  | 1.84 (0.49-6.96) |  |
| Linear trend | 1.19 (0.96-1.46) | 0.105 | 1.13 (0.91-1.41) | 0.257 | 1.06 (0.84-1.32) | 0.628 |

Model^1^ adjusted initially for socioeconomic position, income, home ownership, marital status, maternal education, gender and parity.

Model^2^ further adjusted for tobacco use during 1-3 months of pregnancy, illicit drug use during 1-3 months of pregnancy and maternal depression at 18 weeks gestation.

^b^Omnibus p-value

**Supplementary Table 10: Depression at age 18 and partners alcohol amount at 18 weeks gestation, complete case**

|  | Unadjusted |  | Adjusted^1^ |  | Adjusted^2^ | |  |
| --- | --- | --- | --- | --- | --- | --- | --- |
| *n*=2572 | OR (CI) | *P* | OR (CI) | *P* | OR (CI) | | *P* |
| Never | 1.00 (ref) | 0.128^b^ | 1.00 (ref) | 0.356^b^ | 1.00 (ref) |  | 0.338^b^ |
| <1 glass per week | 1.62 (0.63-4.17) |  | 1.61 (0.62-4.20) |  | 1.63 (0.62-4.27) | |  |
| 1+ glass per week | 1.18 (0.47-2.99) |  | 1.24 (0.48-3.17) |  | 1.27 (0.49-3.27) | |  |
| 1-2 glasses per day | 0.86 (0.32-2.34) |  | 0.97 (0.35-2.69) |  | 0.97 (0.35-2.68) | |  |
| 3+ glasses per day | 1.50 (0.49-4.56) |  | 1.36 (0.44-4.24) |  | 1.24 (0.39-3.89) | |  |
| Linear trend | 0.88 (0.73-1.05) | 0.158 | 0.90 (0.75-1.08) | 0.241 | 0.88 (0.73-1.05) | | 0.163 |

Model^1^ adjusted initially for socioeconomic position, income, home ownership, marital status, maternal education, gender and parity.

Model^2^ further adjusted for tobacco use during 1-3 months of pregnancy, illicit drug use during 1-3 months of pregnancy and maternal depression at 18 weeks gestation.

^b^Omnibus p-value

**Supplementary Table 11: Depression at age 18 and partners alcohol binge at 18 weeks gestation, complete case**

|  | Unadjusted | | Adjusted^1^ | | Adjusted^2^ | |
| --- | --- | --- | --- | --- | --- | --- |
| *n*=2586 | OR (CI) | *P* | OR (CI) | *P* | OR (CI) | *P* |
| None | 1.00 (ref) | 0.515^b^ | 1.00 (ref) | 0.388^b^ | 1.00 (ref) | 0.304^b^ |
| 1-2 days | 1.08 (0.65-1.78) |  | 1.08 (0.64-1.80) |  | 1.05 (0.62-1.75) |  |
| 3-4 days | 1.01 (0.61-1.66) |  | 1.00 (0.60-1.65) |  | 0.97 (0.58-1.62) |  |
| 5-10 days | 1.25 (0.80-1.97) |  | 1.23 (0.77-1.95) |  | 1.19 (0.75-1.89) |  |
| >10 days | 0.84 (0.50-1.41) |  | 0.77 (0.45-1.31) |  | 0.71 (0.42-1.22) |  |
| Linear trend | 0.99 (0.89-1.10) | 0.879 | 0.97 (0.87-1.09) | 0.630 | 0.96 (0.86-1.07) | 0.465 |

Model^1^ adjusted initially for socioeconomic position, income, home ownership, marital status, maternal education, gender and parity.

Model^2^ further adjusted for tobacco use during 1-3 months of pregnancy, illicit drug use during 1-3 months of pregnancy and maternal depression at 18 weeks gestation.

^b^Omnibus p-value

**Supplementary Table 12:** **Depression at age 18 and mother and partner alcohol binge at 18 weeks gestation, imputed data.**

|  |  | Unadjusted | | Adjusted^1^ | | Adjusted^2^ | | Adjusted^3^ | |
| --- | --- | --- | --- | --- | --- | --- | --- | --- | --- |
|  | *n* = 13480 | OR (CI) | *P* | OR (CI) | *P* | OR (CI) | *P* | OR (CI) | *P* |
| Mothers | None | 1.00 (ref) | 0.466^a^ | 1.00 (ref) | 0.708^a^ | 1.00 (ref) | 0.797^a^ | 1.00 (ref) | 0.797^a^ |
|  | 1-2 days | 1.07 (0.76-1.52) |  | 1.00 (0.70-1.42) |  | 0.95 (0.67-1.37) |  | 0.98 (0.68-1.41) |  |
|  | 3-4 days | 1.55 (0.98-2.43) |  | 1.37 (0.85-2.20) |  | 1.22 (0.75-1.98) |  | 1.27 (0.78-2.06) |  |
|  | 5-10 days | 1.19 (0.57-2.48) |  | 1.07 (0.51-2.27) |  | 0.95 (0.44-2.03) |  | 1.01 (0.47-2.16) |  |
|  | >10 days | 0.89 (0.40-1.98) |  | 0.76 (0.33-1.72) |  | 0.68 (0.29-1.57) |  | 0.71 (0.31-1.63) |  |
|  | Linear trend | 1.06 (0.94-1.19) | 0.329 | 1.01 (0.90-1.15) | 0.831 | 0.97 (0.86-1.11) | 0.697 | 0.99 (0.87-1.13) | 0.884 |
|  |  |  |  |  |  |  |  |  |  |
| Fathers | None | 1.00 (ref) | 0.348^a^ | 1.00 (ref) | 0.378^a^ | 1.00 (ref) | 0.354^a^ | 1.00 (ref) | 0.363^a^ |
|  | 1-2 days | 0.85 (0.61-1.19) |  | 0.87 (0.62-1.23) |  | 0.87 (0.62-1.22) |  | 0.87 (0.62-1.22) |  |
|  | 3-4 days | 0.74 (0.53-1.05) |  | 0.76 (0.53-1.09) |  | 0.76 (0.53-1.09) |  | 0.76 (0.54-1.09) |  |
|  | 5-10 days | 0.89 (0.45-1.22) |  | 0.92 (0.66-1.29) |  | 0.91 (0.65-1.28) |  | 0.91 (0.66-1.28) |  |
|  | >10 days | 0.75 (0.53-1.06) |  | 0.75 (0.52-1.07) |  | 0.73 (0.51-1.06) |  | 0.74 (0.51-1.06) |  |
|  | Linear trend | 0.95 (0.88-1.03) | 0.195 | 0.95 (0.88-1.03) | 0.224 | 0.95 (0.87-1.03) | 0.189 | 0.95 (0.87-1.03) | 0.194 |
| Model ^1^: socioeconomic position, income, home ownership, marital status, maternal education, gender, parity  Model ^2^: socioeconomic position, income, home ownership, marital status, maternal education, gender, parity, maternal tobacco use during 1-3 months of pregnancy, maternal illicit drug use during 1-3 months of pregnancy, maternal depression 18 weeks gestation  Model ^3^: socioeconomic position, income, home ownership, marital status, maternal education, gender, parity, maternal tobacco use during 1-3 months of pregnancy, maternal illicit drug use during 1-3 months of pregnancy, maternal depression 18 weeks gestation, how often partner consumed alcohol at 18 weeks gestation  ^a^Wald test | | | | | | | | | |

| **Supplementary material Table 13: Depression at age 24 and mother and partner alcohol amount at 18 weeks gestation, imputed data.** | | | | | | | | | |
| --- | --- | --- | --- | --- | --- | --- | --- | --- | --- |
|  |  | Unadjusted | | Adjusted^1^ | | Adjusted^2^ | | Adjusted^3^ | |
|  | *n* = 13480 | OR (CI) | *P* | OR (CI) | *P* | OR (CI) | *P* | OR (CI) | *P* |
| Mothers | Never | 1.00 (ref) | 0.367^a^ | 1.00 (ref) | 0.729^a^ | 1.00 (ref) | 0.873^a^ | 1.00 (ref) | 0.882^a^ |
|  | <1 glass per week | 0.93 (0.74-1.16) |  | 0.96 (0.76-1.21) |  | 0.94 (0.75-1.19) |  | 0.94 (0.74-1.19) |  |
|  | 1+ glass per week | 1.09 (0.81-1.46) |  | 1.07 (0.79-1.45) |  | 1.02 (0.75-1.39) |  | 1.01 (0.74-1.37) |  |
|  | 1-2 glasses per day | 1.75 (0.84-3.62) |  | 1.55 (0.72-3.31) |  | 1.38 (0.64-2.97) |  | 1.36 (0.63-2.94) |  |
|  | 3+ glasses per day | 1.71 (0.33-8.90) |  | 1.28 (0.23-7.13) |  | 1.09 (0.19-6.15) |  | 1.08 (0.19-6.02) |  |
|  | Linear trend | 1.07 (0.93-1.24) | 0.353 | 1.06 (0.91-1.22) | 0.465 | 1.03 (0.88-1.19) | 0.727 | 1.02 (0.88-1.18) | 0.768 |
|  |  |  |  |  |  |  |  |  |  |
| Fathers | Never | 1.00 (ref) | 0.372^a^ | 1.00 (ref) | 0.684^a^ | 1.00 (ref) | 0.752^a^ | 1.00 (ref) | ^a^ |
|  | <1 glass per week | 0.72 (0.42-1.24) |  | 0.78 (0.44-1.36) |  | 0.78 (0.45-1.37) |  | 0.78 (.044-1.36) |  |
|  | 1+ glass per week | 0.67 (0.39-1.15) |  | 0.79 (0.46-1.37) |  | 0.80 (0.46-1.39) |  | 0.79 (0.46-1.37) |  |
|  | 1-2 glasses per day | 0.69 (0.38-1.25) |  | 0.85 (0.47-1.56) |  | 0.86 (0.47-1.57) |  | 0.84 (0.46-1.54) |  |
|  | 3+ glasses per day | 0.92 (0.48-1.76) |  | 1.03 (0.53-2.00) |  | 1.02 (0.52-1.98) |  | 1.00 (0.52-1.91) |  |
|  | Linear trend | 0.98 (0.86-1.12) | 0.733 | 1.03 (0.90-1.17) | 0.706 | 1.02 (0.90-1.17) | 0.740 | 1.02 (0.89-1.16) | 0.790 |
| Model ^1^: socioeconomic position, income, home ownership, marital status, maternal education, gender, parity  Model ^2^: socioeconomic position, income, home ownership, marital status, maternal education, gender, parity, maternal tobacco use during 1-3 months of pregnancy, maternal illicit drug use during 1-3 months of pregnancy, maternal depression 18 weeks gestation  Model ^3^: socioeconomic position, income, home ownership, marital status, maternal education, gender, parity, maternal tobacco use during 1-3 months of pregnancy, maternal illicit drug use during 1-3 months of pregnancy, maternal depression 18 weeks gestation, how often partner consumed alcohol at 18 weeks gestation  ^a^Wald test | | | | | | | | | |

| **Supplementary material Table 14: Depression at age 24 and mother and partner alcohol binge at 18 weeks gestation, imputed data.** | | | | | | | | | |
| --- | --- | --- | --- | --- | --- | --- | --- | --- | --- |
|  |  | Unadjusted | | Adjusted^1^ | | Adjusted^2^ | | Adjusted^3^ | |
|  | *n* = 13480 | OR (CI) | *P* | OR (CI) | *P* | OR (CI) | *P* | OR (CI) | *P* |
| Mothers | None | 1.00 (ref) | 0.349^a^ | 1.00 (ref) | 0.746^a^ | 1.00 (ref) | 0.823^a^ | 1.00 (ref) | 0.817^a^ |
|  | 1-2 days | 1.03 (0.70-1.51) |  | 0.92 (0.62-1.36) |  | 0.87 (0.58-1.31) |  | 0.88 (0.59-1.31) |  |
|  | 3-4 days | 1.40 (0.88-2.24) |  | 1.21 (0.74-1.99) |  | 1.12 (0.68-1.84) |  | 1.13 (0.69-1.85) |  |
|  | 5-10 days | 1.15 (0.48-2.73) |  | 0.98 (0.40-2.42) |  | 0.91 (0.37-2.24) |  | 0.92 (0.37-2.26) |  |
|  | >10 days | 1.76 (0.94-3.30) |  | 1.46 (0.76-2.81) |  | 1.35 (0.69-2.65) |  | 1.36 (0.69-2.69) |  |
|  | Linear trend | 1.13 (1.00-1.27) | 0.044 | 1.07 (0.94-1.21) | 0.305 | 1.04 (0.91-1.19) | 0.549 | 1.04 (0.92-1.19) | 0.515 |
|  |  |  |  |  |  |  |  |  |  |
| Fathers | None | 1.00 (ref) | 0.727^a^ | 1.00 (ref) | 0.859^a^ | 1.00 (ref) | 0.865^a^ | 1.00 (ref) | ^a^ |
|  | 1-2 days | 0.85 (0.58-1.23) |  | 0.88 (0.60-1.29) |  | 0.88 (0.60-1.29) |  | 0.87 (0.60-1.27) |  |
|  | 3-4 days | 0.79 (0.55-1.12) |  | 0.84 (0.59-1.20) |  | 0.83 (0.58-1.19) |  | 0.82 (0.58-1.17) |  |
|  | 5-10 days | 0.91 (0.66-1.25) |  | 0.97 (0.70-1.35) |  | 0.96 (0.69-1.33) |  | 0.94 (0.68-1.31) |  |
|  | >10 days | 0.93 (0.65-1.33) |  | 0.95 (0.66-1.37) |  | 0.93 (0.64-1.34) |  | 0.90 (0.63-1.31) |  |
|  | Linear trend | 0.99 (0.92-1.07) | 0.847 | 1.00 (0.92-1.08) | 0.989 | 0.99 (0.92-1.08) | 0.872 | 0.99 (0.91-1.07) | 0.771 |
| Model ^1^: socioeconomic position, income, home ownership, marital status, maternal education, gender, parity  Model ^2^: socioeconomic position, income, home ownership, marital status, maternal education, gender, parity, maternal tobacco use during 1-3 months of pregnancy, maternal illicit drug use during 1-3 months of pregnancy, maternal depression 18 weeks gestation  Model ^3^: socioeconomic position, income, home ownership, marital status, maternal education, gender, parity, maternal tobacco use during 1-3 months of pregnancy, maternal illicit drug use during 1-3 months of pregnancy, maternal depression 18 weeks gestation, how often partner consumed alcohol at 18 weeks gestation  ^a^Wald test | | | | | | | | | |

**Supplementary Table 15: Depression age 24 and maternal alcohol binge 32 weeks gestation imputed data**

|  | Unadjusted | | Adjusted^1^ | | Adjusted^2^ | |
| --- | --- | --- | --- | --- | --- | --- |
| *n* = 13480 | OR (CI) | *P* | OR (CI) | *P* | OR (CI) | *P* |
| None | 1.00 (ref) | 0.0004^a^ | 1.00 (ref) | 0.003^a^ | 1.00 (ref) | 0.008^a^ |
| 1-2 days | 1.47 (1.05-2.08) |  | 1.34 (0.94-1.91) |  | 1.29 (0.90-1.84) |  |
| 3-4 days | 1.01 (0.52-1.99) |  | 0.89 (0.45-1.78) |  | 0.84 (0.42-1.68) |  |
| 5-10 days | 1.28 (0.58-2.81) |  | 1.12 (0.49-2.57) |  | 1.05 (0.46-2.41) |  |
| >10 days | 4.66 (2.12-10.27) |  | 4.34 (1.89-9.98) |  | 3.94 (1.69-9.20) |  |
| Linear trend | 1.28 (1.12-1.47) | 0.001 | 1.23 (1.06-1.43) | 0.006 | 1.20 (1.03-1.40) | 0.019 |
| Model^1^ adjusted initially for socioeconomic position, income, home ownership, marital status, maternal education, gender and parity.  Model^2^ further adjusted for tobacco use during 1-3 months of pregnancy, illicit drug use during 1-3 months of pregnancy and maternal depression at 18 weeks gestation  ^a^Wald test | | | | | | |

| Supplementary Table 16: Proportions of missing data for exposures, outcomes and confounders | | |
| --- | --- | --- |
|  | Complete data *n* | Missing data *n* (%) |
| Maternal alcohol frequency 18 weeks gestation | 13195 | 2250 (15%) |
| Maternal alcohol binge 18 weeks gestation | 13149 | 2296 (15%) |
| Maternal alcohol binge 32 weeks gestation | 8780 | 6665 (43%) |
| Partner alcohol frequency 18 weeks gestation | 9841 | 5604 (36%) |
| Partner alcohol binge 18 weeks gestation | 9941 | 5504 (36%) |
| Offspring depression (18 years) | 4563 | 10882 (70%) |
| Socioeconomic position | 10118 | 5327 (34%) |
| Income | 9949 | 5506 (33%) |
| Home ownership | 13501 | 1944 (13%) |
| Marital status | 13559 | 1886 (12%) |
| Parity | 13124 | 2321 (15%) |
| Smoked cigarettes during pregnancy | 13359 | 2086 (14%) |
| Illicit drug use during pregnancy | 13098 | 2347 (15%) |
| Maternal depression 18 weeks gestation | 12161 | 3284 (21%) |
